# The Impact of an Adaptive mHealth Intervention on Improving Patient-Provider Health Care Communication: Secondary Analysis of the DIAMANTE Trial

**DOI:** 10.2196/64296

**Published:** 2025-07-17

**Authors:** Lynn Leng, Marvyn R Arévalo Avalos, Adrian Aguilera, Courtney R Lyles

**Affiliations:** 1 School of Public Health, UC Berkeley-UCSF Joint Medical Program, University of California, Berkeley, 1995 University Avenue, Suite 300, Berkeley, CA, 94704, United States, 1 510 642 5479; 2 School of Medicine, University of California, San Francisco, San Francisco, CA, United States; 3 School of Social Welfare, University of California, Berkeley, Berkeley, CA, United States; 4 Department of Psychiatry and Behavioral Sciences, University of California, San Francisco, San Francisco, CA, United States; 5 Department of Public Health Sciences, Center for Healthcare Policy and Research, University of California, Davis, Davis, CA, United States

**Keywords:** mobile health, mhealth, chronic care management, patient engagement, patient-physician relationship, underserved, depression, diabetes, comorbidity, health literacy, self-management, application, app

## Abstract

**Background:**

Depression and diabetes are highly comorbid conditions and are significant causes of global disability, particularly among individuals with low income or those from racial or ethnic minority backgrounds. While digital interventions offer promise for managing these chronic conditions (such as via lifestyle modification), there is also emerging evidence suggesting that digital support may strengthen or complement existing health care relationships, particularly by improving patient perceptions of communication and connection with their health care providers.

**Objective:**

This study aimed to examine the impact of an adaptive mobile health (mHealth) texting-based intervention on patient ratings of communication with their health care providers among individuals with diabetes and depressive symptoms.

**Methods:**

This study used data from the DIAMANTE (Diabetes and Mental Health Adaptive Notification Tracking and Evaluation) trial, a prospective, randomized controlled trial that varied SMS text messaging strategies to encourage physical activity to support both mental and physical health for patients with diabetes and depression. Patients were recruited from safety-net clinics in San Francisco and through social media during the COVID-19 pandemic, and were randomized into three trial arms: (1) personalized SMS text messaging about physical activity via an adaptive learning algorithm, (2) randomly selected SMS text messaging about physical activity, and (3) a control group that received no SMS text messages. As a secondary outcome, we examined pre-post changes in patient-reported health care communication, assessed via surveys with the validated Consumer Assessment of Healthcare Providers and Systems (CAHPS) communication subscale. Bivariate comparisons examined changes in CAHPS scores, including age, gender, preferred language, race or ethnicity, nativity, marital status, and education. Our primary analysis used mixed-effects modeling within an intent-to-treat analysis to determine differences in CAHPS scores by trial arm.

**Results:**

A total of 195 patients participated in the DIAMANTE trial from 2020 to 2022. After excluding patients who had incomplete or missing baseline or exit surveys, 156 patients were included in this secondary analysis. Overall, there was a substantive but nonsignificant decrease in the average CAHPS score over the 6-month trial period (−2.6; *P*=.11), with similar trends across patient demographic subgroups. Upon evaluating health care communication across the three randomized controlled trial (RCT) arms, there were no significant differences in patient-provider communication.

**Conclusions:**

Digital health interventions are rapidly increasing in clinical practice and have the ability to reach historically underserved communities by overcoming barriers such as language, geography, and time constraints. However, RCTs have primarily focused on the clinical impact of these tools, rather than how the tools may impact patients’ perceptions of their relationships with providers. While our study found no significant changes in patient-provider communication by RCT arm, the temporal implications of the COVID-19 pandemic during the DIAMANTE study period remain unclear and should be further studied. This study is the first of its kind to examine the influence of an adaptive mHealth intervention on patient-reported health care communication, with insights that may be important as digitally-enabled chronic care management rapidly expands.

## Introduction

Treatment and care for depression and diabetes, two highly prevalent and often co-occurring conditions [1,2], are often siloed. Individuals with diabetes have an increased risk of developing comorbid depression compared to individuals without diabetes [3]. This comorbidity is often associated with a worse prognosis for both conditions [4], occurs at higher rates, and results in worse outcomes, particularly in marginalized communities, including individuals with low income, limited health literacy, or from racial or ethnic minority groups [5,6]. Management of chronic diseases such as diabetes requires self-management practices such as maintaining adequate physical activity and adhering to medications [6]. One important factor that affects self-management practices is positive patient-provider communication [7], which has been documented in many studies to increase trust and improve health outcomes among patients with chronic illness [8].

Mobile health (mHealth) interventions have the potential to effectively integrate with existing chronic health management plans. mHealth is defined as “medical and public health practice supported by mobile devices, such as mobile phones, patient monitoring devices, personal digital assistants, and other wireless devices [9].” The advent of cost-effective mHealth apps has been effective in helping patients engage in healthy behaviors, such as increased physical activity and lowered HbA_1c_ levels [10]. For instance, a diabetes SMS text messaging program improved HbA_1c_ scores as well as provided emotional support for patients [11]. Further, the COVID-19 pandemic has expedited the use of telehealth resources and SMS text messaging through secure platforms via electronic devices, which has improved access to health care [12].

The rapidly increasing number of chronic disease mHealth apps indicates the demand for self-management tools [7]; however, there must be a consideration of digital equity in terms of individuals’ internet access, skills, and support related to these platforms and tools [13]. For example, mAdherence tools (ie, mobile health adherence tools) focusing on individuals with low income as well as those who are elderly and from racial or ethnic minority backgrounds were found to be effective and with high satisfaction ratings, although these required a strong baseline adherence program to begin with, as well as active encouragement from the clinical team [14]. Similarly, mHealth tools can be tailored for patients with non-English language preferences; with the addition of culturally concordant motivational messaging, mHealth tools have the potential to reduce health care disparities [15].

Recently, there has also been a surge in mHealth apps integrating artificial intelligence to address chronic health management needs and equity concerns via more personalized communication, such as adaptive learning which tailors messaging in response to an individual’s behavior and characteristics [15,16]. Importantly, personalization of messaging could also reinforce and extend the patient-provider relationship that was already established during a face-to-face interaction. Previous observational studies have found that digital communication and remote monitoring could improve the quality and quantity of existing patient-provider relationships, leading to ‘digital intimacy,’ characterized by familiarity made possible by electronic devices in addition to in-person encounters [17]. This was true even when participants were aware that the digital communication was computer- or digitally-generated [13,17]. While this body of literature is growing, there is a lack of randomized trial evidence about the impact of these interventions on patient-provider communication outcomes.

Therefore, we sought to examine the impact of a personalized communication intervention on participants’ ratings of provider communication during the study period. Specifically, this study is a secondary analysis of an existing RCT evaluating a digital health physical activity intervention using reinforcement learning algorithms among adult patients with depression and diabetes.

## Methods

### Ethical Considerations

This study was reviewed and approved by the Institutional Review Board (IRB) of the University of California, San Francisco (IRB #17-22608). All procedures performed in studies involving human participants were conducted in accordance with the ethical standards of the institutional and/or national research committee and with the WMA Declaration of Helsinki. Informed consent was obtained from all individual participants included in the study. Participants were provided US $110 for completion of the study and all data was deidentified. Further details of the study are described by Aguilera et al [18].

### Study Design

The DIAMANTE (Diabetes and Mental Health Adaptive Notification Tracking and Evaluation) was a prospective, IRB-approved, RCT evaluating the effectiveness of a machine-learning-guided smartphone intervention on physical activity. In brief, the intervention sent SMS text messages to participants during a 6-month period to encourage physical activity to support both mental and physical health [19]. There were three arms of the trial: (1) the control arm received weekly messages to report their mood; (2) the random messaging arm received two additional daily text messages that were randomly selected from a message bank that provided feedback on step counts as well as a motivational message related to being physically active, and (3) the adaptive messaging arm used a reinforcement learning algorithm to personalize these daily feedback and motivational messages for each participant based on their demographics as well as their recent data (eg, their steps taken on the previous day, time elapsed since the previous message type that was sent to them) [19].

Development of the SMS text messaging system was designed using a behavioral theory, and the iterative design was completed with patients in a safety-net health care setting as well as via Amazon Mechanical Turk (MTurk) crowdsourcing [20]. For example, (1) the feedback messages were created to provide positive (eg, “Great job!”), neutral (eg, “You walked 4000 steps yesterday”), and negative (eg, “You can do better!”) feedback and 2) the motivational messages were categorized within theoretical categories of capability (ie, knowledge and skills to be active), opportunity (ie, advice on strategies to be active, both independently and with others), and motivation (ie, cognitive messages about beliefs and emotions that impact health behaviors). The final DIAMANTE intervention tracked participant step counts from pedometers on smartphones, via an application designed in English and Spanish for both Apple App Store and Android Google Play [19]. Further details about the DIAMANTE application design process have been published elsewhere, including details about the reinforcement learning algorithm and the main RCT findings [18].

### Recruitment

English- and Spanish-speaking patients aged 18‐75 years, diagnosed with diabetes and had documented depressive symptoms (score >5 on PHQ-8), and who had an iPhone or Android smartphone were eligible for participation. Exclusion criteria included the inability to exercise due to physical disability, active psychosis or mania, active suicidal ideation, severe cognitive impairment, inability to read and write in English and Spanish, current pregnancy, and plans to leave the country for extended periods during the 6-month trial.

From 2020 to 2022, patients were first recruited from safety-net clinics in the San Francisco Health Network, the public health care system for the city and county of San Francisco. Due to the COVID-19 pandemic, the study team then expanded recruitment to individuals on social media (ie, Craigslist, Facebook, and Google) who met the same eligibility criteria and prioritized major metropolitan areas with diabetes and larger proportions of Spanish-speaking residents [20]. All patients in the San Francisco Health Network were recruited via their existing primary care provider, and all patients recruited online self-attested that their clinician had diagnosed them with diabetes; both parameters facilitated the focus on patient-provider communication in this paper. Patients received a compensation of $40 for participation in the baseline study and an additional $70 for completion of the 6-month active intervention follow-up. Both of the time points included a detailed 50-item survey with numerous validated scales and self-reported data.

### Measures

We examined the impact of the randomized intervention on pre-post changes in patient-reported health care communication via participant surveys at baseline and follow-up, based on the validated Consumer Assessment of Healthcare Providers and Systems (CAHPS) communication subscale [21]. The CAHPS score is widely used across outpatient practices to assess the quality of communication and care processes, and is a well-documented 4-item measure of how often providers explained things well, listened carefully, showed respect, and spent enough time with patients (responses ranged from ‘never’ to ‘always,’ coded from 1 to 4). Using conventional scoring, response items were linearly transformed to aid in interpretation (eg, scores of 1, 2, 3, and 4 correspond to 25, 50, 75, and 100, respectively) and averaged across questions, resulting in a total possible score from 25 to 100, with 25 being the lowest possible score, reflecting poor communication between the patient and provider. This scale is one of the most widely used measures of patient-provider communication in routine clinical practice in the United States [22].

Covariates examined descriptively in this study included (1) age (tertiles of ≤45, 46‐55, or ≥56); (2) gender (ie, man, woman, transgender, and gender queer), (3) education (≤ high school, some college, or ≥ college graduate), (4) race or ethnicity (Asian/Pacific Islander, Black or African American, Hispanic/Latino, White, or Multiethnic), (5) marital status (single, separated, widowed, married, or partnered), (6) born in the United States (US nativity: yes or no), and (7) preferred language (English or Spanish). Covariates were chosen based on previous evidence of their potential impacts on communication outcomes, in core domains of socioeconomic status (eg, education), potential communication barriers (eg, language, nativity), social support (eg, marital status), and other demographics previously associated with communication scores (eg, gender, race or ethnicity).

### Statistical Analysis

We first examined the unadjusted descriptive associations between various demographic measures and changes in CAHPS scores from baseline to exit surveys using paired two-tailed *t* tests. These bivariate comparisons examined changes in CAHPS score based on participant age, gender, preferred language, race or ethnicity, nativity, marital status, and education.

Further, our primary analysis examined the changes in CAHPS scores by RCT intervention arm. As the data were from a randomized controlled trial, we did not adjust for the covariates. We used mixed effects modeling in an intent-to-treat primary analysis to determine any significant differences in the communication outcome based on the intervention arm.

## Results

### *t* Test Findings

Between 2020 to 2022, 195 patients participated in the DIAMANTE trial. After taking account of participants who dropped out (either due to incomplete data or missing baseline or exit survey), 156 patients were included in the analysis. Participants’ mean age was 48.3 (SD 12.2) years and 62.2% (97/195) were predominantly female. A total of 76.3% (n=119) participants were predominantly English-language preferring, were born in the United States (64.1%, n=100), and represented Asian/Pacific Islander (7.7%, n=12), Black or African American (17.3%, n=27), White (28.8%, n=45), Hispanic or Latino (36.5%, n=57), and Multiethnic (9.6%, n=15) racial and ethnic groups. A large portion of participants were single (44.2%, n=92) or married (27.6%, n=64), and had at least some college education and beyond (68.6%, n=107).

As shown in Table 1, there was an overall decrease in the CAHPS scores from baseline to follow-up for most demographic subgroups, indicating communication measures declined during the DIAMANTE trial, although these differences were not statistically significant. This included all categories of gender, age, English and Spanish language, race and ethnicity, US and non-US nativity status, marital status, and most educational attainment levels. A few patterns emerged as well when descriptively comparing groups; greater raw decreases in CAHPS scores were observed among Asian or Pacific Islander and Black or African American participants. In educational attainment levels and age groups, there were larger raw decreases in those who had some college education and less, and those who were younger, respectively.

**Table 1.** Baseline demographic and clinical characteristics.

Variables	Participants (N=156), n (%)	Baseline CAHPS^a^ score (N=156)	Exit survey CAHPS score (N=156)	*P* value
Gender				
Man	55 (35.3)	85.5	84.4	.68
Woman	97 (62.2)	81.2	77.5	.11
Transgender and Gender Queer	4 (2.5)	68.8	68.8	≥.99
Age				
<45	62 (39.8)	80.7	76.1	.15
46‐55	42 (26.9)	84.5	81.7	.31
>56	52 (33.3)	82.6	82.4	.96
Language				
English	119 (76.3)	80.7	77.9	.15
Spanish	37 (23.7)	87.7	85.6	.50
Race/Ethnicity				
Asian/Pacific Islander	12 (7.7)	79.7	71.9	.16
Black or African American	27 (17.3)	89.4	82.4	.13
White or Caucasian	45 (28.9)	76.4	74.7	.62
Hispanic/Latino	57 (36.5)	84.9	83.8	.68
Multiethnic	15 (9.6)	80.4	80.8	.93
Born in the United States				
Yes	100 (64.1)	80.4	77.5	.20
No	56 (35.9)	85.8	83.7	.33
Marital Status				
Single/Separated/Widowed	92 (59.0)	85.9	82.9	.15
Married/Partnered	64 (41.0)	77.8	75.4	.39
Education				
<High School	49 (31.4)	85.2	80.1	.13
Some College	40 (25.7)	86.3	81.7	.10
≥College Graduate	67 (42.9)	78.3	78.0	.91

a CAHPS: Consumer Assessment of Healthcare Providers and Systems.

### Paired Samples Results

Overall, there was a marginal decrease in the average CAHPS score over the DIAMANTE clinical trial period of six months. A mean decrease of −2.6 points (95% CI −5.9 to 0.6; *P*=.11) was observed across the 156 patients who completed both the baseline and exit surveys, which was not statistically significant.

### Mixed-Effects Modeling Results

**Figure 1.**
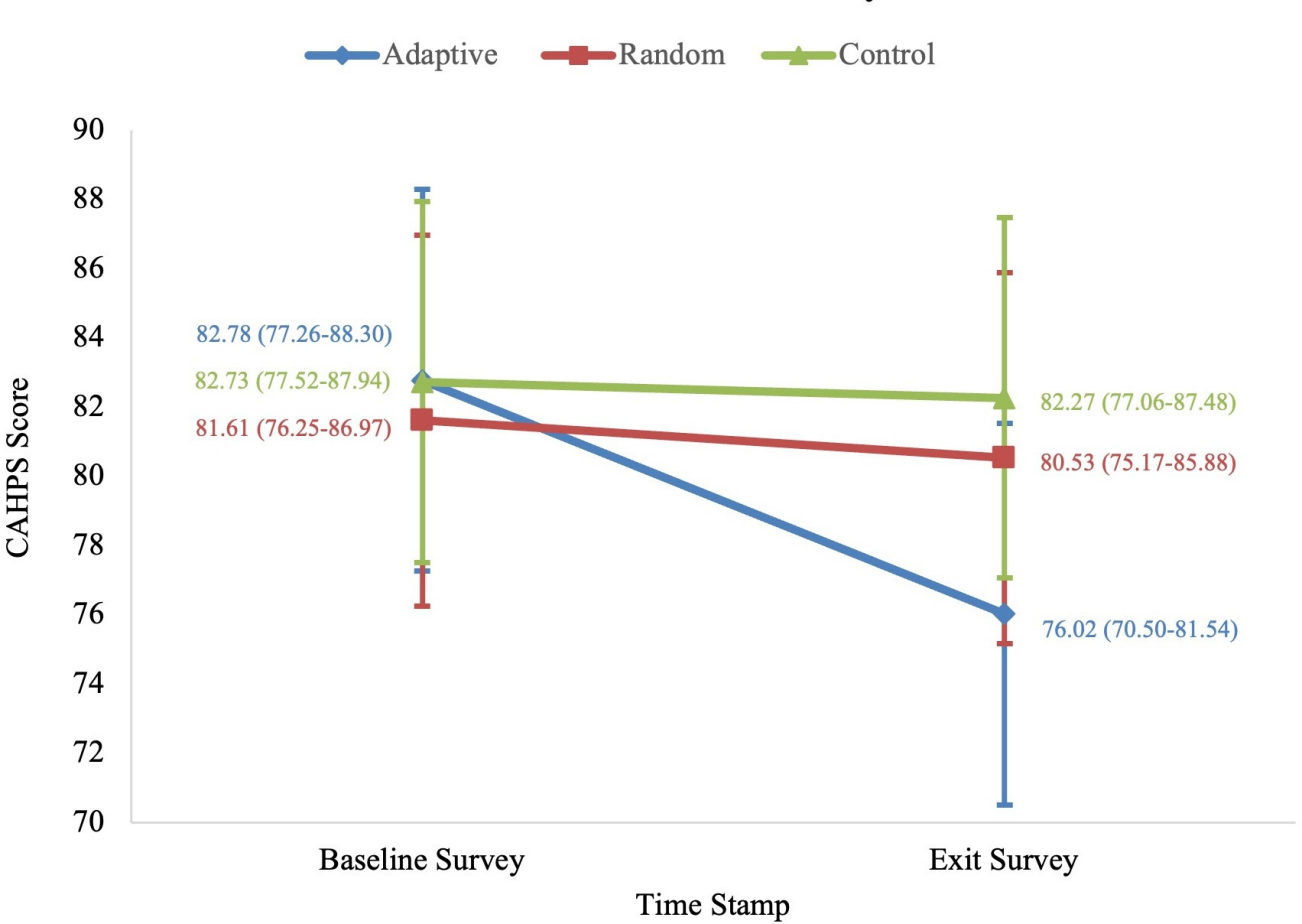
Mixed effects modeling to denote CAHPS score changes by intervention arms. CAHPS: Consumer Assessment of Healthcare Providers and Systems.

Further, in the primary intent-to-treat analysis, there were no significant differences in CAHPS scores between the three intervention arms (Figure 1). While not statistically significant, the largest decrease in CAHPS score was seen in the adaptive arm (−6.3 95% CI −13.8 to 1.3; *P*=.11) compared to the control arm.

## Discussion

### Principal Findings

Our study examined patient-reported health care communication using CAHPS scores over the course of a 6-month RCT study (DIAMANTE). There was an overall marginal decrease in communication scores reported by patients in the study, without any significant differences by intervention arm, suggesting that the intervention did not alter patient-reported communication over time. Findings varied across different demographic subgroups; however, these trends in decreasing communication were similar in most subgroups.

Our findings are contrary to some previous literature which may suggest improvements in communication by leveraging digital technology. Previous studies show that initiating mHealth apps in the context of an already established patient-provider relationship is vital in building effective communication channels [14,23]. Additionally, increased touchpoints and motivational behaviors from providers throughout the usage of the mHealth app have been shown to improve patient-provider communication [24,25] and cultivate a greater sense of connectedness compared to traditional clinical encounters [26]. However, to our knowledge, this is the first evidence specific to RCTs using personalized digital communication.

While our study found no significant relationships between the digital intervention and patient reports of communication, it uncovered overall decreasing trends in patient-provider communication. One major reason for this finding may be related to the influence of the COVID-19 pandemic on overall patient-provider communication. While patient-provider communication ratings measured by CAHPS at a national level have been steadily increasing through 2019, there was a decrease in hospital CAHPS scores over 2020‐2021 period [27]; other findings have been mixed. For example, one previous study found that hospital CAHPS scores decreased across 3381 US hospitals during COVID-19 [28], whereas other studies found that CAHPS scores did not differ between in-person and telehealth visits, with one comparing scores before and during COVID-19 and the second study comparing CAHPS score variations between the two services during the pandemic [29,30]. Thus, it is unclear how the COVID-19 pandemic, which limited in-person interactions between patients and their providers uniformly, impacted the foundational rapport and trust between patients and providers.

Lastly, it has been documented that the pandemic exacerbated burnout among clinicians [28]. This is particularly important given the need for clinicians to have sufficient support and capability to invest in relationship-centered care, and this was particularly challenging in the context of the pandemic with many competing demands (ie, both mental and physical) in health care settings [31]. Additional research has also demonstrated the rapidly increasing digital communication channels and resulting workload on clinicians in recent years [32]. Therefore, there may be potential for mHealth tools, particularly those that are more personalized, to impact patients’ perceptions of their relationship with their health care team via physician workload pathways. Moving forward, more research is needed on how the role of digital communication, including personalized communication with machine learning can support both patient perceptions of their ongoing care as well as the relevance of the technology to the well-being and workflows of clinicians. Further, since many mHealth tools are used for chronic care management or educational support outside the confines of traditional clinical communication, it is important to distinguish the role of these tools on ongoing provider relationships, particularly for patients who have longitudinal care from the same providers.

### Limitations

This study has several limitations. The study sample is limited due to the nature of the original RCT. Although CAHPS is a validated source of assessing patient-provider trust and communication, more granular measures of patient-provider communication, such as assessments of specific types and frequency of communication for chronic illness management, may have identified different patterns or mechanisms related to patient-provider communication. In addition, it was difficult to tease out the role of the COVID-19 pandemic and the multiple recruitment strategies for the DIAMANTE trial, particularly related to variation in the connection between each patient and their primary care provider. However, to our knowledge, this is the first longitudinal assessment of CAHPS communication scores examined within a randomized trial involving a digital intervention, which is an important base for future research. With the surge of artificial intelligence and adaptive learning technologies, there is a need for ethical research on how these techniques affect communication and the trust of the communities whose confidence in the health care system was already fragmented.

### Conclusions

As the use of adaptive learning–based digital health apps have increased across health care facilities, it is important for technology to extend, not replace strong in-person relationships built on trust and evidence–based communication strategies. In-person communication skills will remain critical even as the pace of digital and adaptive technology implementation accelerates.

## Supplementary material

10.2196/64296 Checklist 1 CONSORT-EHEALTH checklist (V 1.6.1).

## Abbreviations

CAHPS: Consumer Assessment of Healthcare Providers and Systems
DIAMANTE: Diabetes and Mental Health Adaptive Notification Tracking and Evaluation
HbA_1c_: glycosylated hemoglobin
mHealth: mobile health
RCT: randomized controlled trial
